# Potential Composite Digenic Contribution of *NPC1* and *NOD2* Leading to Atypical Lethal Niemann-Pick Type C with Initial Crohn’s Disease-like Presentation: Genotype-Phenotype Correlation Study

**DOI:** 10.3390/genes13060973

**Published:** 2022-05-29

**Authors:** Bilal Azab, Omar Rabab’h, Dunia Aburizeg, Hashim Mohammad, Zain Dardas, Lina Mustafa, Ruba A. Khasawneh, Heyam Awad, Ma’mon M. Hatmal, Eyad Altamimi

**Affiliations:** 1 Department of Pathology and Cell Biology, Columbia University Irving Medical Center, New York, NY 10032, USA; 2 Department of Pathology and Microbiology and Forensic Medicine, School of Medicine, The University of Jordan, Amman 11942, Jordan; dunia.aburizeg@gmail.com (D.A.); hashim96mohd@gmail.com (H.M.); limustafa95@gmail.com (L.M.); h_awad@ju.edu.jo (H.A.); 3 Department of Internal Medicine, University of Iowa Carver College of Medicine, Iowa City, IA 52242, USA; omar-rababh@uiowa.edu; 4 Department of Molecular and Human Genetics, Baylor College of Medicine, Houston, TX 77030, USA; zain.dardas@bcm.edu; 5 Department of Diagnostic Radiology and Nuclear Medicine, Faculty of Medicine King Abdullah University Hospital, Jordan University of Science and Technology, Irbid 22110, Jordan; rakhasawneh2@just.edu.jo; 6 Department of Medical Laboratory Sciences, Faculty of Applied Medical Sciences, The Hashemite University, Zarqa 13133, Jordan; mamon@hu.edu.jo; 7 Pediatric Department, Faculty of Medicine, Jordan University of Science and Technology, Irbid 22110, Jordan

**Keywords:** digenic, NPC1, NOD2, Crohn’s, NPC, genotype–phenotype association

## Abstract

Niemann–Pick disease type C (NPC) is an autosomal recessive neurovisceral disease characterized by progressive neurodegeneration with variable involvement of multisystemic abnormalities. Crohn’s disease (CD) is an inflammatory bowel disease (IBD) with a multifactorial etiology influenced by variants in *NOD2*. Here, we investigated a patient with plausible multisystemic overlapping manifestations of both NPC and CD. Her initial hospitalization was due to a prolonged fever and non-bloody diarrhea. A few months later, she presented with recurrent skin tags and anal fissures. Later, her neurological and pulmonary systems progressively deteriorated, leading to her death at the age of three and a half years. Differential diagnosis of her disease encompassed a battery of clinical testing and genetic investigations. The patient’s clinical diagnosis was inconclusive. Specifically, the histopathological findings were directed towards an IBD disease. Nevertheless, the diagnosis of IBD was not consistent with the patient’s subsequent neurological and pulmonary deterioration. Consequently, we utilized a genetic analysis approach to guide the diagnosis of this vague condition. Our phenotype–genotype association attempts led to the identification of candidate disease-causing variants in both *NOD2* and *NPC1*. In this study, we propose a potential composite digenic impact of these two genes as the underlying molecular etiology. This work lays the foundation for future functional and mechanistic studies to unravel the digenic role of *NOD2* and *NPC1*.

## 1. Introduction

Niemann–Pick disease (NP) belongs to the lysosomal storage diseases (LSDs) that are characterized by impairments in the lipid storage compartments. NP is classified into four groups (NP A–D) [1]. So far, there is no approved treatment for NP types A and B. Recently, the treatment of patients with NP-A and NP-B who have neurological involvements with olipudase alfa, an enzyme replacement therapy, is currently under clinical trial [2]. Patients with NP-C and NP-D are treated with miglustat, which has limited efficiency [3]. 

Niemann–Pick disease (NP) type C (NPC) is a progressive, autosomal-recessive lysosomal storage disease manifesting as a neurodevelopmental disorder [4]. NPC can be caused by either *NPC1* or *NPC2* [5]. 

Pathogenic variants in the designated genes affect lipid trafficking, leading to the accumulation of lipid inside the lysosomes. This leads to buildup of lipid-laden macrophages within the brain, liver, lung, and bone marrow [4,5]. Consequently, neurological deficits, restrictive lung disease, aspiration pneumonia, and hepatosplenomegaly may occur [6].

Inflammatory bowel disease (IBD) comprises Crohn’s disease (CD) and ulcerative colitis, both of which are influenced by genetic and environmental factors [7]. *NOD2* encodes a protein triggering an inflammatory response [8]. Several variants in *NOD2* have been shown to cause CD, Blau syndrome (BS), and Yao syndrome (YS) [9]. *NPC1* produces a late-endosomal transmembrane protein that aids in cholesterol trafficking inside the cell [10]. Patients with NPC can experience initial symptoms starting from infancy to adulthood. In addition, the earlier the symptoms manifest, the more severe and progressive the symptoms become [5,11]. Typically, patients with NPC feature infantile hepatosplenomegaly, hypotonia, and a delay in motor milestones [5]. Rarely, they can exhibit features of granulomatous and fistulizing colitis [12]. Noteworthy, the concurrent association between NPC and severe early-onset presentation of CD-like pulmonary manifestations and neurological symptoms has never been previously reported.

Granulomas are considered a form of chronic inflammatory reaction that is characterized by the accumulation of activated epithelioid histiocytes [13]. They occur in two settings: immune responses and foreign body reactions. In immune responses, activated T lymphocytes recruit macrophages via secreting interferon γ [14]. In foreign body reactions, macrophages attempt to phagocytose foreign material that is non-degradable, which results in frustrated phagocytosis and the aggregation of activated macrophages [15].

Here, we report a unique case of NPC showing infantile hepatomegaly, a CD-like picture, and subsequent neurological manifestations. We also highlight, for the first time, the potential role of *NPC1* and *NOD2* in the rapid deterioration of the patient over the course of the disease, which was driven by our phenotype–genotype correlation attempts. We also propose a possible composite digenic effect between *NPC1* and *NOD2*.

## 2. Materials and Methods

### 2.1. Ethical Statement

This study was approved by the institutional review board (IRB) at King Abdullah University Hospital, Irbid, Jordan (approval code: 27/112/2018; approval date: 31 August 2018). All the methods were adherent to the tenets of the Declaration of Helsinki. Informed consent was obtained from the patient’s parents prior to enrolment.

### 2.2. Sample Collection and Sequencing

DNA was extracted from blood samples using QIAprep Spin Mini-prep Kit according to the manufacturer’s instructions. Whole-exome sequencing (WES) was performed as previously described by Azab et al. [16]. Briefly, WES was conducted using the Illumina NovaSeq platform. The reads were aligned to the NCBI reference sequence (GRch38) using the Burrows–Wheeler Aligner. We used a stepwise filtration approach. Only the variants located within the exons and the flanking regions that had a minor allele frequency (MAF) of ≤1% were included. In the first analysis approach, a list of 82 genes known to be associated with IBD was used to conduct the filtration (Table S1). Our second-tier approach was based on filtering all of the WES data for homozygous loss-of-function (LoF) variants (Table S2). The interpretation of the variants was conducted based on the ACMG/AMP guidelines [17]. Co-segregation analysis via Sanger sequencing was conducted for the *NOD2* variant (Supplementary Table S3).

### 2.3. Simulation Analysis

Simulation analyses were deployed to determine the pathogenicity of the identified variants. A template search with BLAST and HHBlits was performed against the SWISSMODEL template library [18,19] for NOD2, truncated NPC1, and CARD9 (a potential binding protein with NOD2) (Supplementary Text S1).

## 3. Results

### 3.1. Overview of the Clinical Presentation

**Figure 1:**
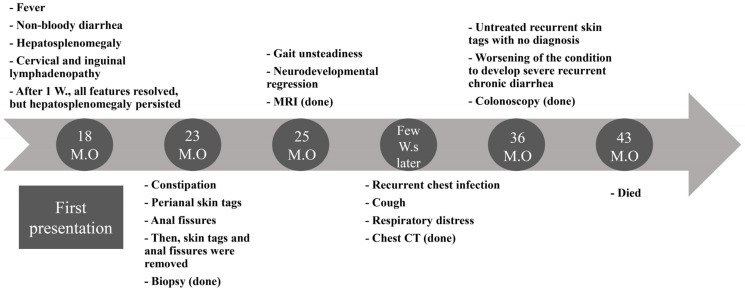
Timeline of the patient’s manifestations.

**Figure 2:**
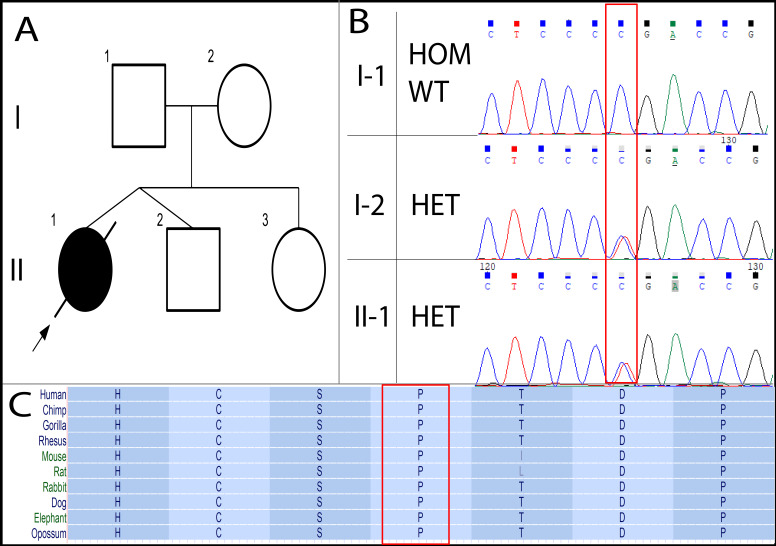
Overview of the patient’s presentation. (**A**) The participating family’s pedigree. (**B**) Chromatograms of the variant in *NOD2* (c.1190C>T). (**C**) The conservation of the amino acid change (p.Pro397Leu) across species from UCSC genome browser. Red rectangles surround the variant.

A three-year-old patient who belonged to a non-consanguineous family was recruited for a genetics investigation. At the time of genetic examination, she had a long history of multi-systemic complaints and negative workup, raising the suspicion of genetic etiology. Both parents and siblings are clinically normal. She was hospitalized multiple times for gastrointestinal, pulmonary, and neurological issues starting at age 1.5 years until she died at 3.5 years (Figure 1 and Figure 2A).

### 3.2. Initial Presentation

She first presented to the emergency room (ER) at 18 months of age with a fever and non-bloody diarrhea for the previous two weeks. On physical examination, she had hepatosplenomegaly, cervical and inguinal lymphadenopathy, and a blanchable skin rash. Her blood investigations showed anemia, thrombocytopenia, atypical lymphocytosis on blood film, and mild elevation in hepatic transaminases. Further workups trying to elucidate an infectious, inflammatory, or metabolic etiology were all negative (Table 1). After one week, diarrhea, fever, and skin rash were all resolved. However, her presentation with hepatosplenomegaly persisted. 

### 3.3. Development of Skin Tags and Anal Fissures and Histopathological Findings

**Figure 3:**
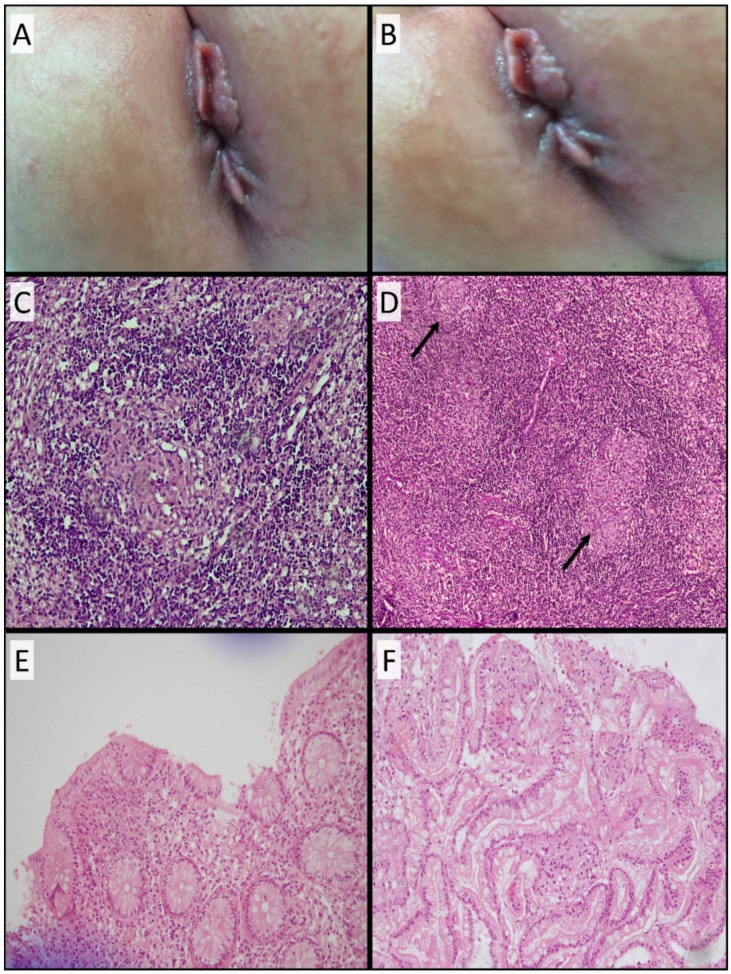
Histopathological description. (**A**,**B**) Patient’s skin tags. (**C**,**D**) Anal fissure and skin tag biopsies showing noncaseating granuloma with heavy lymphohistiocytic inflammation, along with several epithelioid non-caseating granulomas (arrows). These had a thick rim of lymphocytes. (**E**) Colonic biopsy showing normal crypt architecture with no distortion, branching, dropped glands, or basal plasmacytosis. Goblet cells were preserved and there was no evidence of pyloric metaplasia. Chronic inflammatory infiltrate was noted along with a few well-formed epithelioid noncaseating granulomas. These had a thin rim of lymphocytes. Occasional giant cells were identified. (**F**) Ileal biopsy showing normal glandular architecture without granulomas.

Five months later, she presented with multiple non-fistulizing perianal skin tags and anal fissures that did not improve with conservative measures, necessitating surgical removal (Figure 3A,B). Biopsies were taken from the perianal tissues. Surprisingly, her skin’s biopsies (Fissure and Tags) showed mixed inflammatory lymphocytes with several epithelioid non-caseating granulomas. A Zeihl–Neelsen (Z-N) stain was negative, and no acid-fast bacilli were seen (Figure 3C,D). Immunological workups including immunoglobulin subclasses levels, burst test, and flow cytometry were normal. Noteworthy, skin tags kept reemerging over her lifespan. Surgical removal of the skin tags was done multiple times to ease her bowel motions. Colonoscopy was not done at that time due to logistical issues.

### 3.4. Development of Neurological Manifestations and MRI Evaluation

**Figure 4:**
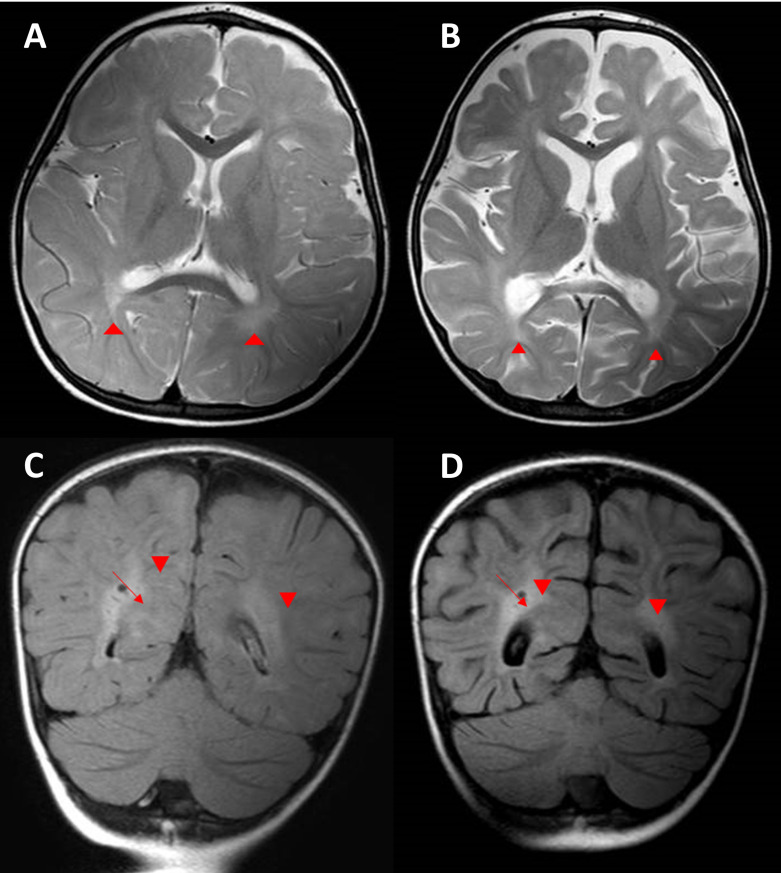
Proband axial brain MRI scans. (**A**) Axial T2 brain MRI showing delayed myelination for the patient’s age with diffuse abnormal high T2 signal intensity seen in the periventricular deep white matter, more prominent in both peri-trigonal regions (arrow heads). There is also prominence of the subarachnoid spaces in both frontal regions. (**B**) Follow-up axial T2 brain MRI demonstrating progressive brain atrophy with unchanged delayed myelination and diffuse abnormally high T2 signal intensity seen in the periventricular deep white matter, more prominent in both peri-trigonal regions (arrowheads). (**C**) Coronal FLAIR brain MRI image demonstrating diffuse abnormally high signal intensity in the periventricular deep white matter, more prominent in both peri-trigonal regions (arrow heads) with prominent VR spaces (arrow). (**D**) Follow-up coronal FLAIR brain MRI demonstrating the progressive brain volume loss with unchanged diffuse abnormally high signal intensity in the periventricular deep white matter, more prominent in both peri-trigonal regions (arrowheads) with prominent VR spaces (arrow).

At the age of 25 months, she started having gait unsteadiness and neurodevelopmental regression. The neurological manifestations were not suggestive of any specific pattern. A brain MRI was performed and showed prominent subarachnoid space in bifrontal regions, abnormal high signal intensity on T2 and FLAIR in both terminal zones, and incomplete myelination noted on T2 involving U fibers of both peri-sylvian regions and high partial regions (Figure 4A,C). The rest of the workups were inconclusive. 

### 3.5. Development of Respiratory Symptoms and Subsequent Neurological Deterioration

After one week, the patient started to complain of pulmonary issues. She presented to the ER with a cough, respiratory distress, and deteriorated consciousness. The patient required admission to the pediatric intensive care unit. Thereafter, she fell into a coma and was intubated secondary to an influenza infection. During her hospital stay, her level of consciousness improved but had worsening neurological deficits after discharge. She had lost her ability to walk and speak and had decreased response to visual and auditory stimuli. She then had seizures and ophthalmoplegia. Regarding worsening neurological function, the brain MRI was repeated and showed progressive loss of brain volume (Figure 4B,D). 

### 3.6. Recurrent Chest Infections and Chest CT Findings 

**Figure 5:**
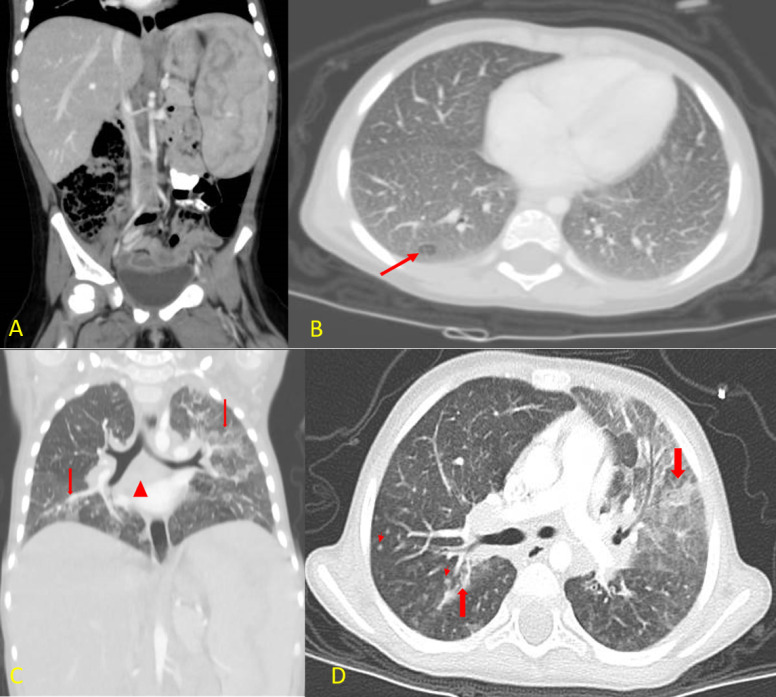
Patient’s CT scans in both coronal (**A**,**C**) and axial (**B**,**D**) views. (**A**) Contrasted CT scan of the abdomen and pelvis showing hepatosplenomegaly. (**B**) Axial lung window settings for lung bases demonstrating diffuse ground-glass opacity of both lung fields with diffuse smooth interstitial thickening and mosaic pattern of lung attenuation (arrow). (**C**) Coronal CT reconstruction demonstrating again the areas of consolidation, ground-glass opacity, and smooth interstitial thickening in both lung fields (thick arrows). The mediastinal lymphadenopathy, especially in the subcarinal region (arrowhead). (**D**) Axial CT scan of the chest with IV contrast lung window settings demonstrating multiple areas of consolidations (thick arrows) with surrounding ground-glass opacity, which were more prominent in the left upper lung lobe. There is also diffuse smooth interstitial thickening, and multiple small intraparenchymal lung nodules can be seen (short arrows).

A few weeks later, she presented repeatedly with recurrent chest infections requiring hospitalization, which were believed to be secondary to recurrent aspiration. She underwent fundoplication with no improvement. She underwent bronchoscopy, and the lavage was negative for acid-fast bacilli. In addition, a PCR and culture for Mycobacterium were negative. Her chest CT showed patchy areas of consolidation and ground-glass opacities seen in both lungs, predominantly in the left upper lobe. Multiple innumerable nodules scattered in both lungs and multiple enlarged lymph nodes in the retro-tracheal, bilateral paratracheal, hilar, and subcarinal were also noted (Figure 5).

### 3.7. Colonoscopy and Histopathological Findings

At the age of three years, the patient developed severe chronic bloody diarrhea. No infectious cause was identified. Then, the patient underwent a colonoscopy, which showed a severely inflamed left colon, scattered aphthous ulcers in the transverse colon, and normal right-sided colonic mucosa. Macroscopically, features were consistent with IBD, specifically CD. Microscopically, colonic biopsies showed normal crypt architecture with no distortion, in addition to a chronic inflammatory infiltrate, along with a few well-formed epithelioid non-caseating granulomas. These had a thin rim of lymphocytes. Occasional giant cells were identified (Figure 3E). The ileal biopsies were normal with no granulomas (Figure 3F). The granulomas in all the specimens were negative with a Z-N stain for acid-fast bacilli and a PAS stain for fungi. 

### 3.8. Treatment Regimen

The patient started on polymeric formula with mild improvement in diarrhea. Methylprednisolone 2 mg/kg was added to her treatment regimen to induce remission, with no significant improvement. The patient kept running fevers with significant diarrhea and high inflammatory markers. The patient was started on anti-TNF α. After the first dose of infliximab, she showed improvement in diarrhea and fever. The patient was not compliant with the treatment regimen due to insurance issues, and her primary response was lost soon after that. 

### 3.9. Dilemma of Establishing Definitive Differential Clinical Diagnosis 

Our initial investigations, based on ordering biochemical and immunological workups to look for any potential culprits or congenital immunodeficiencies, were negative (Table 1). Based on the clinical and pathological findings, the differential diagnoses included an early onset of the CD-like features with extraintestinal manifestations, sarcoidosis, chronic granulomatous disease, BS, and infections. Therefore, the clinicopathological findings were inconclusive for both the chronic interstitial pulmonary changes seen on a chest CT scan and the neurological deficits. Hence, genetic analysis was done to find a genetic etiology. Sadly, her condition further deteriorated, and she died at the age of three and a half years, secondary to respiratory failure.

### 3.10. Genetic Findings 

After conducting a stepwise WES analysis, we revealed potential disease-causing variants (DCV) in two candidate genes, each of which has possible overlapping symptoms with the patient’s clinical presentation. 

Initially, we identified a heterozygous missense variant (c.1190C>T; p.Pro397Leu) in *NOD2*. This change can lead to the substitution of Pro397 with Leu. It has a rare allele frequency in the gnomAD database without any report of homozygosity. There is no consensus on *in silico* prediction for this variant. The position of this sequence change is conserved among various species (Figure 2C). In addition, ClinVar (ID: 531599, last accessed 1 February 2022) has an entry for this variant with conflicting interpretations of uncertain significance (VUS) and benignity. To our knowledge, this variant was not described in the literature (Table 2). Co-segregation analysis via trio-based Sanger sequencing showed that the mother and father harbored the variant’s heterozygous and wild-type forms, respectively (Figure 2B). Given together, the current pieces of evidence are inconclusive to draw a clear pathogenic/benign classification of this variant. Therefore, we classified this variant as a VUS. Further functional studies, reported cases, or co-segregation analyses are needed in order to upgrade the classification to (likely) pathogenic.

Since *NOD2* can be implicated in the development of granulomatous disease, we considered this variant as a potential cause of our patient’s granulomatous presentation. However, the patient’s clinicopathologic picture did not fit the typical concurrent manifestations of *NOD2*-related diseases. Specifically, the proband’s accompanying pulmonary and neurologic involvements had never been described in any patients harboring variants in *NOD2*. Interestingly, the pattern of neurological and pulmonary deterioration could be indicative of another underlying etiology. Therefore, we re-conducted the WES analysis to screen out rare LoF homozygous variants. 

Consequently, we were able to identify another putative frameshift DCV (c.352_353delAG; p.Gln119ValfsTer8) in *NPC1*. This variant results in deleting two nucleotides of the transcript (AG) at positions c.352_353. Consequently, the reading frame of the resulting transcript will be disrupted, creating a premature termination signal after eight codons. The nearest exon–exon junction is >55 bases downstream, suggesting the occurrence of the nonsense-mediated decay (NMD) process. Therefore, this variant could lead to either an absent or truncated protein product. The heterozygous state of this variant has an AF of zero and two out of 251,408 in gnomADv3 and gnomADv2, respectively. Homozygous and compound heterozygous forms of this variant have been reported in patients with NPC [4]. Furthermore, this variant has a consensus pathogenic classification in ClinVar (ID: 370143, last accessed 1 February 2022) by five independent submitters. Altogether, we classified this variant as pathogenic (Table 2). 

The patient’s overlapping clinical synopsis of hepatosplenomegaly, ataxia, developmental regression, and interstitial lung disease can be explained by the context of this variant in *NPC1*. However, our patient presented with a combination of aggressive manifestations that had never been described in patients with NPC. These manifestations include early-onset CD-like condition, pulmonary involvement, and neurological deterioration—implying that the identified variant in *NPC1* could not solely comply with the overall concomitant clinical manifestations in our patient. To elucidate the putative effect of these mutated genes on the corresponding protein level, we resorted to simulation analysis. 

### 3.11. Simulation Analysis of NOD2 and NPC1

**Figure 6:**
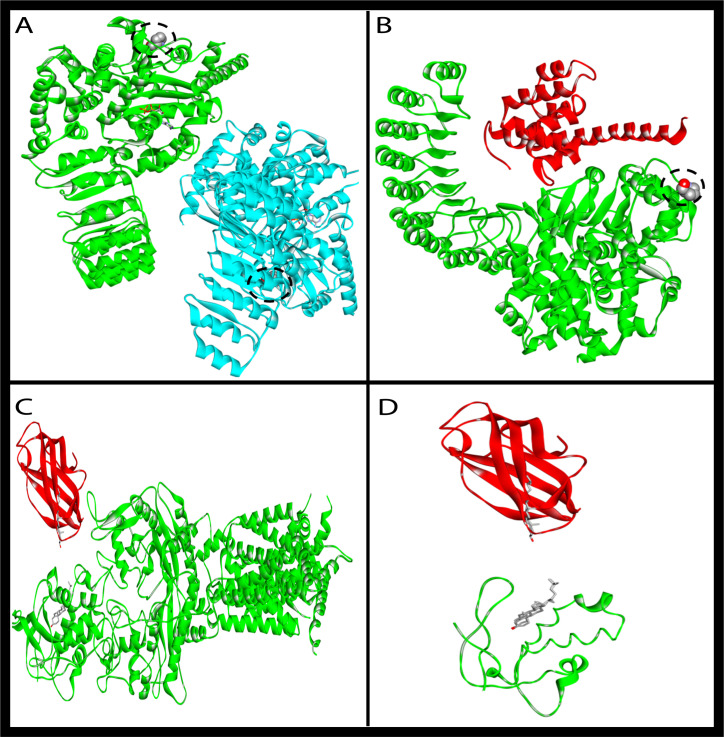
Mutated protein simulation analysis. Illustration of (**A**) NOD2 dimer (green and cyan colors) and (**B**) the best score docked pose of NOD2 monomer (green) and its binding partner CARD9 (red). Proline380 is denoted by space-filling spheres and is surrounded by a dotted circle; it is located far from the binding interface. The NOD2 structure was based on the PDB structure with the ID 5IRM. The variant of proline380 to leucine had no effect on the binding of the two monomers in the dimer form or on the binding with CARD9. Comparison between (**C**) wild-type NPC1 (green) and (**D**) the truncated form of NPC1, and their binding with their partner NPC2 (red). Truncated NPC1 lost its binding interface with NPC2.

The “Calculate Mutation Energy (Stability)” and the “Calculate Mutation Energy (Binding)” revealed that NOD2 proline380 to leucine had a neutral effect on stability and binding with the ligand (ADP), respectively, for both monomer and dimer forms (Figure 6A,B). NOD2 was then employed either as a monomer or as a dimer, and the mutation energy was calculated for the best 10 docked NOD2-CARD9 poses, which had a neutral effect. These findings reveal that NOD2’s proline380 to leucine had no influence on its stability, ligand binding, or binding with putative partner CARD9 (Figure 6A,B). Moreover, superimposing truncated NPC1 on the NPC1-NPC2 complex reveals that truncated NPC1 lost its binding interface with NPC2 (Figure 6C,D).

## 4. Discussion

Here, we describe a patient with an ambiguous clinical presentation of CD-like deterioration. Her worsening condition encompassed neurological deterioration and pulmonary involvement. A battery of clinical, laboratory, histopathological, and imaging studies was ordered, and the diagnosis remained inconclusive. The patient’s clinical features could not fit completely into any of the previously described diseases. Therefore, a genetic approach was pursued to solve the dilemma of reaching the proper diagnosis. Our phenotype–genotype-driven analysis via WES enabled us to identify two candidate genes (*NOD2* and *NPC1*) that could comply with her concomitant presentation. 

On her initial admissions, the patient’s early presentation of fever, diarrhea, and perianal non-caseating granulomatous skin tags raised the suspicion of dealing with an infantile CD disease as the underlying etiology. Given her heterogeneous presentation, we followed a stepwise approach in her medical workups. Initially, we ordered conventional biochemical tests and imaging studies. The biochemical testing included enzyme activity assays for screening various LSDs, such as NP type A and NP type B. The requested enzyme activity assays are considered a suitable method for screening LSDs. However, they are not comprehensive for screening all types of LSDs [20]. These tests were negative and, therefore, ruled out considering the screened LSDs as an underlying disease etiology. 

The histological findings showed the skin tags, anal fissures, and colonic biopsies to have indistinguishable non-caseating granulomas (Figure 3). The granulomas lacked any unique, distinctive characteristics to pinpoint the causative disease. They, however, exhibited a thick lymphocytic rim, which can be seen in various immunologic reactions. Nevertheless, the described lymphocytic rim occurs more frequently in BS than in CD [21]. Sarcoidosis could be a possibility, but no lung biopsies were performed. Sarcoidosis is more commonly found in the respiratory system and hilar lymph nodes than in the gastrointestinal tract [22]. Overall, the histopathological findings were inconclusive to reveal the patient’s underlying granulomatous disease. 

Variant in *NOD2*. We resorted to genetic investigation via WES to unpuzzle her inconclusive findings. First, we analyzed a list of genes known to be associated with infantile IBD (Table S1) and identified a candidate heterozygous missense DCV in *NOD2*. Mono-allelic pathogenic variants in *NOD2* have been associated with BS. Both mono-allelic and bi-allelic variants in *NOD2* have also been linked to increasing susceptibility to developing either CD or YS [9,23]. Co-segregation analysis showed that this variant was maternally inherited (Figure 2B). We did not rule out this variant based on these findings, as incomplete penetrance has been observed in families with variants in *NOD2* [24]. For instance, a BS case was described to harbor a paternally inherited heterozygous variant (p.Glu383Lys) in *NOD2*. Co-segregation analysis revealed that her father, paternal aunt, and siblings were all heterozygous for the same variants. Only her paternal aunt developed the disease, whereas the rest of her family did not [25]. 

NOD2 consists of three domains: the C-terminal leucine-rich repeat domain (LRR), the central nucleotide-binding and oligomerization domain (NOD), and the N-terminal recruitment domain [9]. Variants in the NOD domain have been reported to cause BS, whereas variants in the LRR domain cause CD [8]. The missense variant identified in our patient is in the NOD domain. This variant was reported in ClinVar in a patient with BS. Although the variants’ locations within NOD2’s domains can be suggestive of the associated disorders, discrepancies have been recorded [26]. 

The preserved crypt architecture, the absence of features of chronicity, the abundance of granulomas, and the thick lymphocytic rim are against the patient’s diagnosis of CD (Figure 3). Moreover, perianal fissures are usually a late feature of CD, and this patient presented with fissures even before the gastrointestinal symptoms. Those findings do not support the differential diagnosis of CD. Nonetheless, those histological findings do not rule out the diagnosis of BS. Notably, the patient did not present with the typical clinical triad of BS, including arthritis, dermatitis, and uveitis [8]. Therefore, we propose that this variant in *NOD2* is implicated in causing a granulomatous disease that is most consistent with an infantile CD-like disease. Whether this patient would have developed the full-blown, previously described clinical picture of CD or not was limited by the short lifespan of the proband. Simulation analysis of mutated NOD2 did not predict any effect on its binding to the ligand or the protein’s stability. However, this does not eliminate the plausible effect of this variant on the protein’s function with either untested or undescribed protein partners. Noteworthy, the proposed role of the mutated *NOD2* in driving the clinical picture of the patient was based on a genotype–phenotype association, rather than mechanistic experiments. We pave the way for additional experimental studies to elucidate the phenotypic and functional effect(s) of the identified variant (p.Pro397Leu) in *NOD2*. 

Worsening condition. Later, unexpectedly, she developed a progressive multi-systemic presentation, including neurodevelopmental regression, interstitial lung disease, recurrent chest infections, and ataxia. Furthermore, the patient’s clinicopathological picture did not fit the typical concomitant symptoms of *NOD2*-related disorders. Particularly, the associated pulmonary and neurologic involvements had never been recorded in individuals with variants in *NOD2*. This raised the possibility of dealing with either an atypical systemic CD-like picture or another undiagnosed disease. Hence, we re-analyzed her WES to delineate any underlying etiology that could explain her concomitant manifestations. As our second-tier approach, we filtered her exome to search for candidate rare homozygous LoF variants.

*NPC1* DCV. We identified a pathogenic frameshift variant in *NPC1*. DCVs in this gene cause NPC, an atypical neurovisceral type of LSD [5]. Patients with NPC show heterogeneous clinical presentations and variable ages of onset, ranging from the perinatal period to adulthood. The differential diagnosis of NPC is challenging since the routine clinical and laboratory tests are within the normal range, which was the case in our patient [27]. 

Previous reports of this variant (p.Gln119ValfsTer8) described the clinical picture of the affected patients; their age of diagnosis with NPC (depending on the presentation of neurological-related symptoms) varied from the infantile to the juvenile period. Nonetheless, none of them displayed similar clinical characteristics to our patient. Specifically, the CD-like presentation and the subsequent pulmonary manifestations had never been reported with this variant [28,29,30,31,32]. Simulation analysis was not previously conducted on the NPC1 harboring this variant. The findings of our simulation analysis show that NPC1 would have lost its interaction with NPC2 if it had escaped the NMD process. This can drastically impact the protein’s function and stability.

IBD in NPC patients. The association between NPC and either an IBD or CD-like picture has rarely been described in literature [12,33,34,35,36]. The development of IBD-like features in patients with *NPC1* variants has been attributed to impairments in autophagosome activity while handling intracellular invading bacteria [12]. A study by Schwerd et al. reported the mean age of IBD diagnosis in 14 NPC patients to be 12.8 ± 8.6 years, whereas our patient’s age of onset was 1.5 years. In contrast to our study, all these cases were characterized by the development of NPC-related features prior to the emergence of IBD [12]. Furthermore, our proband’s gastrointestinal manifestations presented before the emergence of any neurological deficits or pulmonary symptoms. The patient’s gastrointestinal manifestations encompassed perianal granulomatous skin tags, anal fissures, and colitis. The youngest NPC case described by Schwerd et al. was 3.6 years old and showed manifestations of unclassified IBD associated with growth failure, proximal hepatic flexure–ulcerative colitis, diarrhea, and iron-deficiency anemia [12]. Another case by Dike et al. presented a 2-year-old patient with NPC and CD-like symptoms who also showed perianal skin tags in the absence of any neurological or pulmonary manifestations [33]. Our proband showed the earliest (18 M.O.) and most severe presentation compared to the previously described cases. Interestingly, the association between the early manifestations of a CD-like picture, pulmonary involvement, and neurological deterioration presented here had never been described before. This might be attributed to the presence of the *NOD2* variant, which possibly played a role in aggravating the phenotypic impact of the NPC presentation. More functional studies are needed to show the impact of *NOD2* on worsening the clinical picture of the affected subjects.

Pulmonary manifestations in NPC patients. The variant in *NPC1* can also explain the emergence of the proband’s pulmonary symptoms. Pulmonary manifestations are commonly seen in patients with NP type B. In contrast, respiratory system involvement is rare in patients with NPC [37]. Furthermore, pulmonary involvement with NPC has been mostly attributed to *NPC2*, rather than *NPC1* [38]. The significance of *NPC1* in pulmonary sequelae is underestimated and has rarely been reported [39]. Patients with NP might exhibit a wide spectrum of pulmonary-related features. Asymptomatic to recurrent cough, exertional dyspnea, frequent respiratory infections, and respiratory failure are among the described pulmonary involvements [39]. Our patient showed ground-glass opacities in a chest CT and had recurrent episodes of aspiration pneumonia. Her BAL fluid analysis did not show lipid-laden macrophages, which are commonly detected in NPC-affected patients. Unfortunately, neither lung biopsy nor autopsy was done for this patient. Our findings highlight the need to evaluate *NPC1* as a candidate etiology in the management of comparable ambiguous pulmonary symptoms. Prior to our investigation, the association between early-onset CD-like features, pulmonary involvement, and neurological deterioration had not been described in the literature in any patient with a DCV in *NPC1*. 

Interaction between *NPC1* and *NOD2*. The context of the variant in *NPC1* explains the patient’s overlapping clinical description of hepatosplenomegaly, ataxia, developmental regression, and interstitial lung disease. Nevertheless, our patient displayed these features at an earlier stage, with a more aggressive and prominent presentation than ever described before. This may raise the possibility that her severe presentation may not be solely caused by *NPC1* and might be aggravated by the presence of the *NOD2* variant. The identified variant in *NPC1* can contribute to increasing the risk of developing CD and granulomas. Mutated forms of *NPC1* have been shown to impede bacterial clearance by interfering with autophagosome formation, resulting in persistent inflammatory stimulation [12]. Moreover, the variant we identified in *NOD2* is located within the NOD domain and can lead to hyperactivation of the NFkb signaling pathway. Therefore, this promotes the secretion of inflammatory mediators, resulting in a stimulated inflammatory response [40]. Hence, the two variants we identified in *NOD2* and *NPC1* can independently lead to the activation of the immune system. Theoretically, their coexistence could elicit a stronger immune response than *NPC1* alone. This may hasten the development of early granuloma and CD-like diseases. These arguments could support a potential composite digenetic effect of both *NPC1* and *NOD2* [41]. Further studies are needed to establish the phenotypic impact of harboring a combination of variants in both *NPC1* and *NOD2*. Notably, we are presenting a phenotype–genotype correlation study that can be supported or refuted based on future functional or descriptive investigations.

## 5. Conclusions

In conclusion, this work presents a descriptive genotype–phenotype correlation study that lays the foundation for the potential composite digenic effect of both *NOD2* and *NPC1*. This suggested digenic effect could have played a role in aggravating the patient’s clinical presentation. Although NPC can explain the early manifestation of IBD-like disease, the patient exhibited a more severe course with an earlier age of onset than typical NPC cases. Those atypical manifestations included severe pulmonary involvement, progressive decline in neurological function, and prominent gastrointestinal complications. Our findings can aid future research by guiding functional investigations to understand the impact of the proposed digenic effect of *NOD2* and *NPC1*.

## Figures and Tables

**Table 1:** Results of lab tests conducted during the patient’s hospital admissions.

Test Category	Requested Lab Test	Result
Serology	Pathogen (CMV, HBV, HCV, Parvo virus, EBV, Widal and Brucella, TB, HIV, HSVI, HSVII, flu A, flu B and H1N1)	Negative
	C3 and C4	Normal
	Rheumatoid factor (RF)	Normal
	Antinuclear antibodies (ANA)	Normal
Stool analysis	WBC	Elevated
	Reducing substances	Positive
	Infectious agent	Negative
Metabolic workup	Amino acid analysis and organic acid analysis	Normal
	Multiplex newborn screening test (Pompe, Fabry, mucopolysaccharidosis type 1 Krabbe, Gaucher and Niemann–Pick disease type A/B)	Normal
Immunology workup	Immunoglobulins levels	Normal
	Flow cytometry	Elevated CD19 B cells
	Burst test	Normal
Miscellaneous tests	LDH	Elevated
	Ferritin	Elevated
	α fetoprotein	Normal
	B-HCG	Normal
	CK	Normal
	Lipid profile	Low HDL

**Table 2:** Description of the detected variants.

Gene	Variant Coordinate	Transcript ID	Exon	Variant Description				*In-silico* Prediction
				dbSNP ID	HGVS cDNA aa	Zygo	Max AF gnomADv3 gnomADv2	PROVEAN REVEL SIFT (Score)
*NOD2*	hg38:chr16:50711101hg19:chr16:50745012	NM_022162.2	4/12	rs150078153	c.1190C>T p.(Pro397Leu)	HET	V3: 0.0003087 V2: 0.0002711	Deleterious (−5.88) Benign (0.4399) Damaging (0.000)
*NPC1*	hg38:chr18:23568933hg19:chr18:21148897	NM_000271.4	4/25	rs759075595	c.352_353delAG p.(Gln119ValfsTer8)	HOM	V3:0 V2: 0.00004620	-

Ms, missense; Fs, frameshift, HET, heterozygous; HOM, homozygous; Zygo, zygosity; Max AF, maximum allele frequency.

## Data Availability

All the relevant data are provided in the article’s Supplementary Material.

## Supplementary Materials

The following supporting information can be downloaded at: https://www.mdpi.com/article/10.3390/genes13060973/s1, Supplementary Text S1: Simulation analysis detailed methodology [42,43,44,45,46,47,48,49]; Table S1: List of IBD-related genes; Table S2. Analysis steps according to the second-tier approach; Table S3. Primers and conditions used for co-segregation analysis by Sanger sequencing.
