# Supplementary material for: Potential Composite Digenic Contribution of NPC1 and NOD2 Leading to Atypical Lethal Niemann-Pick Type C with Initial Crohn’s Disease-like Presentation: Genotype-Phenotype Correlation Study
Source: Genes (Basel). 2022 May 29;13(6):973. doi: 10.3390/genes13060973 (PMC9223108; doi:10.3390/genes13060973)
Supplement: Supplementary file 1 [file genes-13-00973-s001.zip › genes-1635074-supplementary.pdf]

Potential composite digenic contribution of NPC1 and NOD2 leading to atypical lethal Niemann-pick type C with initial Crohn's disease-like presentation

Bilal Azab[1,2,*], Omar Rababa[3,†], Dunia Aburizeg[2,†], Hashim Mohammad[2], Zain Dardas[4], Lina Mustafa[2], Ruba A. Khasawneh[5], Heyam Awad[2], Ma'mon M. Hatmal[6], Eyad Altamimi[7**]

1- Department of Pathology and Cell Biology, Columbia University Irving Medical Center, New York, NY 10032, USA
2- Department of Pathology and Microbiology and Forensic Medicine, School of Medicine, The University of Jordan, Amman 11942, Jordan
3- Interdisciplinary graduate program in genetics, University of Iowa, Iowa 52242, USA
4- Department of Molecular and Human Genetics, Baylor College of Medicine, Houston, TX, 77030 USA
5- Department of Diagnostic Radiology and Nuclear Medicine, Faculty of Medicine King Abdullah University Hospital, Jordan University of Science and Technology, Irbid 22110, Jordan
6- Department of Medical Laboratory Sciences, Faculty of Applied Medical Sciences, The Hashemite University, PO Box 330127, Zarqa 13133, Jordan
7- Pediatric Department, Faculty of Medicine, Jordan University of Science and Technology, Irbid 22110, Jordan.

† **O.R. and D.A.** Contributed equally to this work.

***Corresponding author:** Dr. Bilal Azab, Department of Pathology and Cell Biology, Columbia University Irving Medical Center, New York, NY 10032, USA. E-mail: ba2659@cumc.columbia.edu

****Corresponding author:** Dr. Eyad Altamimi, Pediatric Department, Faculty of Medicine, Jordan University of Science and Technology, PO Box 3030, Irbid 22110, Jordan. Email: emaltamimi@just.edu.jo

Supplementary Text S1

Simulation analysis detailed methodology

Models were built based on the target-template alignment using ProMod3[1]. Coordinates that are conserved between the target and the template are copied from the template to the model. Insertions and deletions are remodeled using a fragment library. Sidechains are then rebuilt. Finally, the geometry of the resulting model is regularized by using a force field. In case of loop modeling with ProMod3 fails, an alternative model is built with PROMOD-II[2].

The global and per-residue model quality has been assessed using tools implemented in SWISSS-MODEL including QMEAN (Qualitative Model Energy Analysis) score and MolProbity score[3]. The former is a composite of 6 energy values within the homology model matrix related to protein nativeness with score values $\leq$ -4.0 indicating poor quality homology models. The latter, on the other hand, combines several protein parameters including clash score, Ramachandran Plot criteria (Ramachandran Favored and Ramachandran Outliers)[4].

The "Calculate Mutation Energy (Stability)" protocol in Discovery Studio (version 4.5) was used to evaluate the effect of mutation (NOD2 Proline380 to Leucine) on protein stability. It performs combinatorial amino-acid scanning mutagenesis on a set of selected amino-acid residues by mutating each of them to one or more specified amino-acid types. The energy effect of each mutation on the protein stability (mutation energy, ΔΔGmut) is calculated as the difference of the free energy of folding between the mutated structure and the wild-type protein[5]:

$$\Delta\Delta G\text{mut} = \Delta\Delta G\text{fold(mutant)} - \Delta\Delta G\text{fold(wild type)}$$

Also, the "Calculate Mutation Energy (Binding)" protocol was used to evaluate the effect of mutation (NOD2 Proline380 to Leucine) on the binding affinity of molecular partners in protein-protein and protein-ligand complexes. It performs combinatorial amino-acid scanning mutagenesis on a set of selected amino-acid residues by mutating them to one or more specified amino-acid types. The energy effect of each mutation on the binding affinity (mutation energy, ΔΔGmut) is calculated as the difference between the binding free energy in the mutated structure and wild type protein[5,6]:

$$\Delta\Delta G\text{mut} = \Delta\Delta G\text{bind(mutant)} - \Delta\Delta G\text{bind(wild type)}$$

To Predict the optimal docked poses for two protein structures (NOD2 wild type and CARD9), HDOCK is used to dock the two proteins. It is a fully integrated set of tools for robust and quick protein–protein docking that includes homology search, template-based modeling, structure prediction, macromolecular docking, biological information inclusion, and task management. The service predicts receptor and ligand interactions based on input information for receptor and ligand molecules (either amino acid sequences or Protein Data Bank structures) using a hybrid method of template-based and template-free docking[7].

The "Calculate Mutation Energy (Stability)" protocol in Discovery Studio (version 4.5) was used to determine the stability of the 10 best docked poses upon mutating NOD2 Proline380 to Leucine.

The Superimpose-Molecular overlay tool in Discovery Studio (version 4.5) is used to superimpose truncated NPC1 on the NPC1-NPC2 complex, and the superimposed complex is examined with the same software. NPC1 and NPC2 proteins are required for lysosomal cholesterol egress, and their defects cause Niemann-Pick disease type C (NPC)[8].

The simulation analysis focused on understanding the effects of both variants on protein structure and function. The resulting homology structure was evaluated using structure assessment tools within SWISS-

MODEL. The point mutated NOD2 was prepared using Discovery Studio (version 4.5). Both wild-type and mutated proteins (either in the monomer or dimer forms) were minimized for 10 steps using Discovery Studio (version 4.5).

References:

1. A W, M B, S B, et al. SWISS-MODEL: homology modelling of protein structures and complexes. *Nucleic Acids Res*. 2018;46(W1):W296-W303. doi:10.1093/NAR/GKY427
2. N G, MC P, T S. Automated comparative protein structure modeling with SWISS-MODEL and Swiss-PdbViewer: a historical perspective. *Electrophoresis*. 2009;30 Suppl 1(SUPPL. 1). doi:10.1002/ELPS.200900140
3. CJ W, JJ H, NW M, et al. MolProbity: More and better reference data for improved all-atom structure validation. *Protein Sci*. 2018;27(1):293-315. doi:10.1002/PRO.3330
4. SC L, IW D, WB A, et al. Structure validation by Calpha geometry: phi,psi and Cbeta deviation. *Proteins*. 2003;50(3):437-450. doi:10.1002/PROT.10286
5. Spassov VZ, Yan L. pH-selective mutagenesis of protein–protein interfaces: In silico design of therapeutic antibodies with prolonged half-life. *Proteins*. 2013;81(4):704. doi:10.1002/PROT.24230
6. Spassov VZ, Yan L. A fast and accurate computational approach to protein ionization. *Protein Sci*. 2008;17(11):1955-1970. doi:10.1110/PS.036335.108
7. Yan Y, Tao H, He J, Huang SY. The HDOCK server for integrated protein-protein docking. *Nat Protoc*. 2020;15(5):1829-1852. doi:10.1038/S41596-020-0312-X
8. Qian H, Wu X, Du X, et al. Structural Basis of Low-pH-Dependent Lysosomal Cholesterol Egress by NPC1 and NPC2. *Cell*. 2020;182(1):98-111.e18. doi:10.1016/J.CELL.2020.05.020

Table S1. The list of IBD-related genes

ADA	*DCLRE1C*	*LIG4*	*SH2D1A*	*IL17RA*
ADAM17	*DKC1*	*LRBA*	*SI*	*IL23R*
AICDA	*DOCK8*	*MEFV*	*SKIV2L*	*IL7R*
ANKZF1	*DUOX2*	*MVK*	*SLC37A4*	*LYST*
ARPC1B	*FCHO1*	*NCF2*	*STAT1*	*NHEJ1*
BACH2	*FOXP3*	*NCF4*	*STAT3*	*ORAI1*
BTK	*G6PC3*	*NFAT5*	*STIM1*	*PNP*
C17orf62	*ICOS*	*NLRC4*	*STXBP2*	*PRF1*
CARD8	*IL10*	*NOD2*	*TGFB1*	*PRKDC*
CARMIL2	*IL10RA*	*PIK3CD*	*TGFBR1*	*PTPRC*
CD3G	*IL10RB*	*PIK3R1*	*TGFBR2*	*RFX5*
CD40	*IL21*	*PLCG2*	*TTC37*	*RFXANK*
CD40LG	*IL2RA*	*POLA1*	*TTC7A*	*RFXAP*
CD55	*IL2RB*	*RAG1*	*WAS*	*STX11*
CTLA4	*IL2RG*	*RAG2*	*XIAP*	
CYBA	*ITGB2*	*RIPK1*	*ZAP70*	
CYBB	*JAK1*	*RTEL1*	*ZNF341*	

Table S2. Analysis steps according to the second-tier approach

Applied filter	Number of variants
**Total number of variants**	117,527
**Variants with total read depth of ≥10x**	105,284
**Exonic and flaking variants**	15,042
**LoF variants**	3,621
**Variants with subpopulation MAF of ≤ 1%***	185
**Homozygotes****	13

* According to the following databases: GnomAD, GnomADExome, TOPMed, 1000 Genomes Project, NHLBI Exome Sequencing Project, Global Minor Allele Population Frequency, DGV, 1000 Genomes

** List of genes (# of variants): *NPC1* (1), *FCGBP* (9), *MUC4* (1), *MUC19* (2)

Table S3. Primers and conditions used for co-segregation analysis by Sanger sequencing

Gene	Forward primer	Reverse primer	Amplicon size	Tm.
NOD2	AACCACTCTCTGTGCGGACT	CAGAGAAGCCCTTGAGGTTG	324	60
